# Bethlem Hospital in 1852

**Published:** 1853-01-01

**Authors:** 


### Art. III.—BETHLEM HOSPITAL IN 1852.

The late investigation into the management of Bethlem Hospital imposes upon us a most painful task, a most onerous duty. Willingly—most gladly—would we close our eyes to these sad and melancholy revelations, throw the pen aside, and drop the curtain upon the scene; but the time has arrived when, as journalists, we can no longer postpone the fulfilment of an imperious and necessary duty, however repugnant its performance may be to our own feelings. The professional and the public mind have been shocked by accusations of systematic cruelties, violence, and neglect, in a metropolitan institution, governed by the leading city magistrates. The disclosures that have been made, rise in their enormity, when it is remembered that the governors of Bethlem had all the advantages attending an historical experience of many centuries, to warn them of error, and to guide them to improvement; ample funds to carry out whatever projects their own knowledge, or the progress of medical science, might suggest, with no limitations of their power, no control which could in any way embarrass either their deliberations or their acts; in short, every possible facility—every possible means—to enable them to develop and adapt their institution to the benevolent purposes which it was implicitly entrusted to them to execute. Experience, money, uncontrolled authority—nothing has been wanting to enable the governors of Bethlem to make this institution in reality that which their president vainly boasted that it was—a "model asylum." But how different is the truth! An inquiry, which we are bound to consider an impartial one, by the Commissioners in Lunacy, has terminated in convicting the officials and servants of the asylum, of neglect the most shameful, of cruelties the most revolting; and in the unreserved condemnation of the whole system of government and management, as inefficient and mischievous! Before the public and the profession, Bethlem at this moment appears as a foul blot upon the character of the one, and a scandal to the other. Public decency has been outraged; and the reputation of the medical profession in this department, the most interesting, has been rudely shaken. To allow an institution where such laxity and confusion have prevailed in the government, and where the patients have been subjected to such lamentable treatment, to be represented as a model asylum, would expose both the national character to the reproach, and our professional intelligence and humanity to the contempt, of foreign countries. It is impossible to encounter the risk of a censure so degrading and injurious. We cannot, therefore, permit a false delicacy of feeling, or any sentiment of deference to the position of the authorities of Bethlem, to prevent us from giving such a review of the leading facts which have been proved before the Commissioners, or from making such reflections, as we feel to be necessary to place this painful subject in the proper light, and to avert unjust conclusions.

We may premise a proposition which the governing and official authorities may possibly dispute, but which the subsequent narrative will amply justify—that Bethlem has never enjoyed, among the medical profession, or the public, a very high repute among the lunatic asylums of this country. Of late years, when strenuous efforts have been made to improve the condition of the insane, and when, consequently, the public mind has gradually become better informed as to the possibility of treating lunatics upon principles of humanity and science, those few persons who had been prompted by benevolence or by special circumstances, to visit the wards of Bethlem, could not but contrast what they had there witnessed, with the admirable order, and comfort, and cheerfulness, which they had observed in other asylums. Rumours that many things required a vigorous intervention from without, had for some time prevailed; and these rumours were not impaired in force by the well-known facts—that Bethlem enjoyed a special immunity from all public inspection; and that within its walls were confined a large proportion of so-called "criminal lunatics," and of those individuals who were confined as insane, under the direction of the Secretary of State.

At the end of the year 1850, there occurred an event which, among other things, directed official attention to the management of this institution. The history of this case is detailed in the Commissioners' report. A lady was admitted on the 6th of October, and discharged on the 27th of December, of that year. Her sister on her first visit perceived that she had a black eye; on the second visit, she had been removed from an upper storey into the basement ward—a place specially appropriated to dirty and refractory patients. She complained of excessive cold, of having no sheets or blankets, and of sleeping on loose straw; and also of coarse, violent, and abusive conduct from the nurses under whom she was placed. Under such circumstances, it is not surprising, that when she was removed from Bethlem this unfortunate lady's health was seriously injured. Her back is described as having been a mass of sores; she had a large sore on each hip; her body was emaciated, and her legs swollen. A medical man who saw her after her removal doubted whether she would live twenty-four hours. It was found possible, however, when she had somewhat rallied, to take her to the Northampton asylum. On her arrival there, Dr. Nesbitt verified the former particulars as to her bodily condition, and ascertained that she was also labouring under severe prolapsus of the uterus and anus; and that she was quite unconscious of the calls of nature. Her hair was matted, and entangled, and filled with vermin. Such was the result of this poor lady's confinement for less than three months in Bethlem. At Northampton, under judicious medical treatment, kindness, and the requisite personal comforts, she recovered her mental and bodily health, and was restored to her friends perfectly well, at the expiration of between five and six months.

The Commissioners remark upon this case, that although Miss A. M. was in bad health, and under medical treatment by Dr. Monro and the resident apothecary, Dr. W. Wood, there was *no record thereof in the case-book of the hospital!*

Another case, that of Miss H. H., if possible evincing a still greater degree of reckless neglect, occurred after the foregoing, and an investigation being pertinaciously sought by the father, the proper machinery was at length set in motion; and the Commissioners in Lunacy, who, under the act, have no direct authority to visit Bethlem, received a special order from the Home Secretary to make the requisite inquiry, and to report to him the result.

The report which resulted from the inquiry made by the Commissioners was presented to Sir George Grey, in February, 1852. It was held, for the time being, to be "confidential." But, after a time, it was communicated to the Committee of Governors of Bethlem. Hints, however, transpired, that the conduct of the medical officers was seriously impeached. The medical officers became anxious to see the evidence and the report, to enable them to meet the charges that had been mooted. It was held that the report was "confidential," and could not be communicated to them. On the 30th April, a letter, signed by Drs. Monro, Sir A. Morison, and Wood, was addressed to the Committee of Bethlem. It stated the fact, that immediate changes in the medical staff were in contemplation. It submitted, that if those changes had been determined upon in consequence of the report of the Commissioners, the medical officers had a right to be informed what the report was, and what was the evidence upon which it was founded. In that letter, the medical officers "confidently challenged comparison between the condition of Bethlem Hospital and any similar institution;" and they observed, that "mechanical restraint had been gradually diminished, and at last altogether discontinued." They received a reply, intimating that the contemplated changes in the medical staff had been determined upon solely in consequence of the independent and long-antecedent conviction of the governors; and that they had no authority to give the physicians a copy of the report and evidence. A period of agitation followed; criminations and recriminations ensued; letters from one or two of the medical officers were addressed to the public journals. Sir Alexander Morison directed his solicitors to apply to the Home Secretary for the report. The Home Secretary was inexorable. Sir A. Morison then published his grievances in the advertising columns of the daily papers. Questions were asked about the affair in Parliament. During all this time, it was presumed that the governors were preparing their reply. There is some reason to suppose, however, that they were industriously setting their house in order, dismissing implicated keepers, and remodelling the medical staff—of course, in execution of convictions long entertained, and not at all urged on by recent events, or the distinct recommendations in the Commissioners' report. It was no doubt sagaciously resolved, that there should be no hurry in making their reply; public excitement might cool down if a little time were gained; and when their reply appeared, how much greater effect it would have, if the governors could point to the reforms they had carried out, and the actual improved state of the asylum! About October last, however, one of the strictly "confidential" copies of the evidence and the Commissioners' report, by some mysterious means fell under the cormorant grasp of the morning press.* The whole has been remorselessly published; and so the public has been prematurely put in possession of the authentic details. The case thus divulged, questions were made concerning the intentions of the governors, in the Court of Common Council, and in the Court of Aldermen. In the latter place, the president stated that the reply, which he promised should be satisfactory, was forthcoming; but it was not until the 29th November that it was presented to Mr. Secretary Walpole. That, too, is at present "confidential," and will probably not reach the public eye before the publication of our journal.

We possess, however, abundant materials for arriving at an accurate judgment as to the past management of Bethlem, and the measures

* The spirit and manly independence exhibited by the proprietors and editor of the *Daily News*, in ferretting out and publishing this evidence, entitle them to the warmest thanks of every Christian and benevolent man.

required to secure the proper treatment of the inmates, and the rights of the public, for the future, in the lengthened evidence taken by the Commissioners in Lunacy, and their calm and comprehensive report. No impartial person can deny—although, indeed, the late resident medical officer has threatened indictments against some of the witnesses for conspiracy and perjury, a course, however, which it does not appear that he has yet determined upon—that the conclusions advanced in that report are amply substantiated by evidence. In the governors' defence, therefore, we can only look for something in the nature of explanations: possibly they may succeed in extenuating their own share in the particular transactions which form the principal part of the report; possibly they may show that the chief blame should be cast upon the officers; but certainly we cannot entertain the hope that they will rebut any of the precise accusations established, or that they will make any satisfactory *reply*.

The elaborate nature of the report of the Commissioners (which we print in full) will render it unnecessary for us to enter with minuteness into all the details of the investigation. Such points as we think have been dwelt upon with sufficient clearness in that document, we shall pass over lightly, directing our chief attention to those questions which do not come within its scope, and which, in our opinion, demand special consideration.

In estimating the fitness of the government and regulations of an institution to accomplish the end sought to be attained, it is essential, in the first place, to clearly define its objects. A prominent feature of Bethlem is the exclusion of all epileptics, and of all but recent, and supposed curable cases of insanity. The effect of this regulation has been, to render the acute, or recent cases the largest and most important section of the patients. There is a second class, consisting of such of the first class as have passed into what is presumed to be an incurable stage. A third class is composed of what are called "criminal lunatics," and others confined by order of the Home Secretary. Bethlem is in fact the state-asylum. Such are the classes of patients whose care and treatment constitute the special charge of the hospital. In addition to this, the immediate object, it has been held desirable to make the hospital available for the diffusion of a practical knowledge of the nature and treatment of lunacy. How far are the constitution and regulations adapted to accomplish these important purposes? Wherein have they been defective and calculated to defeat them?

The government of Bethlem is confided to a perpetual body of governors, chiefly recruited by the members of the higher ranks of the corporation of the city of London. Indeed, Mr. Alderman Copeland remarked, that the Court of Aldermen were hereditary governors of

Bethlem. The important office of president falls, we believe by usage, to the Lord Mayor at the time being, when a vacancy occurs. In addition to the accession of governors from this source, the president, the treasurer, and those who have served the office of stewards, have the privilege of appointing or nominating a certain number of governors annually. The president, who is the chief ruler and governor, and whose influence is paramount, can *appoint* two governors annually. It is not difficult to conceive that the exercise of such a power might, in the course of a few years, confer upon the president an almost absolute authority. Out of the general body a general committee of forty-two governors and the president and treasurer, are intrusted with the conduct of the affairs of the hospitals of Bridewell and Bethlem. This committee is arranged into six sub-committees—viz., one to audit the accounts, one for the country estate, one for the conduct of the London estate, one to inspect the prisons, one for the House of Occupations, and one which is called the Bethlem sub-committee. It is with the last that we are especially concerned. The general committee is summoned to meet every month at Bridewell hospital. The Bethlem sub-committee is summoned every week, and is the only one which meets at Bethlem hospital. The treasurer is next in rank and influence to the president. His powers are extensive and singularly anomalous. He is at once an officer, with executive duties to discharge, and he occupies the second place among the ruling authorities, whose duty it is to see that the executive duties are properly performed. The following passages from the evidence of Mr. Johnson, and the rules and orders, show the nature of his office, and his power of control over the internal arrangements of the hospital. His duties are "to superintend the affairs of the hospital, both as to its attendants and the details;" to hire at his discretion all attendants, nurses, and other servants, and to dismiss them when it shall appear to him necessary. "Directly he has no power to rescind any rules, but in cases of emergency he takes upon himself to do so." We shall presently draw attention to a remarkable instance of the exercise of this dictatorial power. The medical staff consists of two physicians, a surgeon, and an apothecary (the resident medical officer). The medical staff is assisted, or more correctly speaking, thwarted and embarrassed by a steward and a matron, having independent and conflicting powers. The two physicians are charged with the duty of making an annual report of the state of the medical department. One of them is required to attend at the hospital every day. Each is to visit every part of the hospital. They are to assist the sub-committee with their advice in admitting and discharging patients. They are allowed to take pupils, and are charged with the duty of making the hospital available for the purposes of instruction. The surgeon attends in strictly surgical cases; and has to inspect the bodies of those who die in the hospital. The apothecary is under the immediate control and direction of the president, treasurer, and committee, and is responsible for the due performance of all the regulations of the governors. He is to examine and record the state of the male patients on admission, and to obtain from the matron similar reports of the state of the female patients. He is to observe the instructions of the physicians as to the treatment of the patients, and in their absence to be responsible for the same. When visiting the female side, he is to be always accompanied by the matron, or one of the nurses. He has, besides this, the medical charge of the House of Occupations, and the duty of dispensing and administering all medicines. The steward is to report to the treasurer any instances of neglect or misbehaviour on the part of the attendants. He is to recommend, in conjunction with the apothecary, to the treasurer, all the male attendants to be hired. The steward and the matron have power to give leave of absence to the male and female attendants respectively. The matron is to be under the control of the president, treasurer, and committee. She is to keep books, recording all the instances of restraint, or liberation from restraint, which may take place each day *by her direction.* She is to see that the female patients be taken care of; and to take care that the straw on which the unclean patients are laid, be changed when damp or dirty. She is to take particular care that such of the female patients as are unfit to be at large, be confined to their sleeping apartments, and no person suffered to come to them but in company with a female attendant.

Arrangements so contradictory and so opposed to what has been found necessary to the good government of lunatic asylums, could bear no fruit but discord among the officials, disorder and neglect among the subordinate attendants. As if to make confusion worse confounded, new regulations and instructions, contradicting the printed rules, were liable to be issued, and the treasurer could dispense with all rules, and could, by word of mouth, take patients from the power of the medical officers, and hand them over to a subordinate, *without so much as mentioning the matter to the principals.* Accordingly, it is with no surprise that we find in the evidence, accusations on the part of the governors and treasurer, against the medical officers; and that it is impossible, out of the conflicting statements made, to define and limit, with any degree of precision, the respective duties of the officers. We think it necessary, however, to trace, with what precision we can, the position of the physicians and the apothecary. Mr. Johnson says, very importantly, "I expect more from the physicians than I get!" One of them is there about an hour per day three times a week; on Fridays, when they attend the committee, it takes them two hours and a half. They go through the galleries: it is impossible to go into all the rooms. Mr. Peter Northall Laurie, an influential and active governor, says, "Dr. Monro's attendance has been made by me the subject of complaint. I am not satisfied with the medical treatment; it is of the most slovenly character. Until the year 1843 there was no report presented to the governors. I then drew up a report; but we found it impossible to extort one from the physicians. We have now got the physicians' report; I call it a turnpike-gate road-list of those who come in and go out; but as to its being a valuable report, it is neither creditable to the hospital nor of value." With regard to the abolition of restraint, Mr. N. Laurie says, "The physicians resisted it, and reported against it. At last, however, I threatened, if they did not do it to the extent it has been reduced in other establishments, I would take means of giving publicity to that state of things. I then found it was done." Such is the account given of the degree of attention the physicians devoted to their charge. The evidence of the same governor conveys the imputation of a dereliction of duty, if possible still more culpable. Mr. N. Laurie says, in reference to the distinction between the moral and medical treatment of the patients, "I consider that *direct medical treatment forms but a small portion of the treatment in a lunatic hospital;* that in the moral treatment must be placed the greatest confidence; and *that can only be properly superintended by a resident medical officer.*" Need we insult the understandings of our readers by refuting the monstrous assertion of Mr. Laurie respecting "direct medical treatment"? But what was the conduct of the treasurer, in reference to this, the all-essential part of the treatment of the patients? He thus explains his views of the functions of the resident medical officer: "His duty is to go round the hospital frequently to see all the patients; *not particularly to interfere with their moral treatment,* but to be with them a great deal." When called upon to explain what he calls the moral treatment, he defines it as follows: "To see that the patients are properly employed, and take proper exercise." This, the moral treatment, the treasurer assigned to the matron; the medical officer having the power to *suggest* what he might consider essential.

Such being the moral treatment of the patients, and the provision made for carrying it out, let us see what the medical treatment consisted of. The almost entire absence of medical case-books or records, renders it difficult to ascertain what kind of medical treatment the patients were in the habit of receiving; unless, indeed, we are to infer from that circumstance that there was little or no medical treatment to record. Accordingly, we find one of the governors characterizing it as slovenly in the extreme. The Commissioners state in their report, that the physicians did little beyond assisting at the admission and discharge of patients, except occasionally prescribing in serious cases. There cannot, surely, be a more convincing proof of the utter want of interest taken in the medical treatment, than the fact so argumentatively insisted upon by the Commissioners—viz., that in this richly-endowed royal hospital, containing upwards of 400 patients, there was no infirmary—no place where the sick could be received and attended apart from the rest! But for the fact that an infirmary is *now* actually being prepared or completed, we should not have been surprised to hear the governors and the physicians contending that they had gone on very well without an infirmary for three hundred and fifty years, and denying that the patients had ever suffered in consequence. We suppose, but for this fact, we should find them contending that an infirmary was perfectly unnecessary, on the plea urged by Dr. Monro—viz., that "all patients who are so sick as to require the attendance of nurses are inadmissible; and when they become sick, they are sent to their relatives. It is a great advantage to go into the country, and that is often suggested in cases of consumption." There is one proposition here which will hardly be disputed. It *is* a great advantage for a sick patient in Bethlem to go into the country, or anywhere else, for escape. The case of Miss A. M., who recovered on being removed to Northampton, proves the advantage of going into the country in a very decided manner. The same case illustrates another singular characteristic of Bethlem medical practice. Miss A. M. was suffering from severe prolapsus uteri, attended by a profuse discharge. There can be no doubt that a disease of this kind required active medical treatment; but although it appears that she was prescribed for by Dr. Monro, the prolapsus, undoubtedly the most serious of her bodily ailments, was *not reported* to Dr. Monro, and *therefore* he was not bound to know anything about it. According to the Bethlem medical creed, prolapsus is an "*infirmity*," and should have been reported to the *surgeon!* The etiquette of the past century is beautifully preserved. But, in sober seriousness, what is the prospect of recovery for a patient surrounded by such an unhappy complication of medical, surgical, and matronly advice as poor Miss M? A physician for her medical relief, a surgeon for her "infirmity," a matron for her "moral treatment and to give her clean straw," an apothecary to attend to her when nobody else did, and yet no one responsible!

We could not give a complete representation of the management of the patients, without showing the kind of treatment they are subjected to from the keepers. We presume this treatment is something distinct in the mind of the treasurer from either medical or moral treatment. The bedding, if such it may be called, in the back basements, devoted to wet and dirty female patients, consists of loose straw placed in a trough, and covered with a blanket. Upon this the patient is laid in a state of complete nudity. In the instance already adverted to, that of Miss A. M., this treatment was proved to have caused sores and excoriations on different parts of the body. She was habitually assailed with opprobrious epithets by the nurses, but over that, the matron says she could have no control. The case of Miss Hyson, also referred to in the report, is still more deplorable. She died in a few days after her discharge from the asylum—an event accelerated, it can scarcely be doubted, by the effects of utter neglect and shameful brutality. The very questionable practice of fastening down reluctant patients in the restraint-chair, and forcing down food by means of the stomach-pump, was commonly resorted to. In order to accomplish the task of dressing the refractory patients, it is deposed, that a stocking was applied round their throats and tightened after the fashion of a tourniquet, in order "to exhaust them."

With the exception of the employment of the stomach-pump, which was the duty of the apothecary, we are informed that governors, president, treasurer, matron, physicians, resident medical officer, and matron, were totally ignorant of the practices described; a disavowal not less disgraceful to all than would have been the plain confession that such things had been done and continued with their knowledge and approbation. The disavowal of all cognizance of these facts involves the self-accusation of the grossest neglect of duty, neglect which nothing can excuse. How can we believe that the physicians complied with that plain instruction in the rules, "to visit every part of the hospital," and at the same time understand how they could fail to see that female patients were littered down naked in straw? We are informed, that they did not know it; we are, consequently, bound to conclude that they neglected their obvious duty. But is it credible that all this should have been unknown to the apothecary, who had been seven years resident in the hospital, and whose duty it was daily, once or oftener, to visit the wards? If we are compelled to accept testimony upon oath, what must be the amount of our astonishment at hearing so shameless a confession of supineness and incompetency! Can we imagine how officers who, on their own showing, had been so systematically remiss in the discharge of their duty, have been so long retained in their positions? Can the governors themselves escape from the similar dilemma of guilty knowledge, or equally guilty ignorance? How can they who had industriously procured by legislative enactment the exclusion of all other inspection, excuse their own neglect?

But leaving to the physicians, the apothecary, and the governors, to select the alternative they may think the least damaging to themselves in this respect, we cannot forbear making some observations upon the conduct of those concerned in reference to the assignment of the moral treatment of the female patients to the matron, leaving to the apothecary, the immediate representative of the physicians, the simple power of "suggesting" to that functionary what he might consider desirable. We have before seen that this change was made on the sole verbal authority of the treasurer, who did not even think it necessary to report the step he had taken to the committee. But if the treasurer never doubted the sufficiency of his own authority, is it possible that it was never called in question by the physicians whose province was thus insultingly invaded? They, too, have under the rules the power of "suggesting reformation;" and we presume there was nothing to prevent an appeal against such a flagrant outrage to themselves and to us all. It cannot be urged for a moment, that the interference of the treasurer was simply between the apothecary and the matron. The female patients were the patients of the physicians, and the apothecary, not only by usage, but by the express rules of the hospital, was their representative and deputy. "He is to observe the instructions of the physicians;" "he is to report to the physicians any alterations he may have made." And shall we be told, in this instance too, that the physicians were uninformed of the change that had been made? When questioned upon this point, Dr. Monro asserts, that *he did not remember having heard of the treasurer's order upon this subject;* but he admits that he was aware that it was the matron's practice to classify patients as she thought proper. Surely the physicians must then have remonstrated—have protested indignantly against such a practice. They could not supinely surrender the direct control over their patients to a matron. They could not passively acquiesce in a measure insulting to themselves, degrading to their profession, and fraught with destruction to the unhappy beings under their charge.

We have not had access to the minutes of the proceedings of the Bethlem Committee: the terms in which the protest of the physicians was couched must therefore remain unknown to us. Nor are we informed as to the mode in which the committee replied. But it does appear, from the evidence of the treasurer, that he transferred the most important part of the treatment of the female patients to the matron, now more than three years ago; and we have not heard that any step was taken to restore the conduct of the moral treatment to the physicians. The extraordinary power conferred upon the matron in 1849, was still, by her own account, exercised by her in 1851; and the physicians had not resigned.

Those of our readers who are conversant with the nature of insanity will not need to be reminded, that the classification, employment, and amusements of lunatics, and the other influences embraced under the term "moral treatment," constitute almost the entire duty of the physician. What is embraced under the Bethlem definition of "medical treatment" is comparatively unimportant as an agent of cure. It is unnecessary to say, that the conduct of the moral treatment of the insane demands a far more profound scientific and practical skill in the higher departments of medicine, than does the mere administration of the remedies of the pharmacopœia. We are well assured that few medical officers of our county asylums would feel it consistent with their personal respect, and still less with their sense of duty to their patients, to retain their appointments under such regulations as prevailed at Bethlem.

Having thus taken a summary view of the recent condition of Bethlem, we now think it useful to draw attention to the last occasion on which it was made the subject of public inquiry. The parallel will suggest many striking and instructive reflections. In the year 1815, a committee of the House of Commons was charged with the duty of examining into the condition of the madhouses in England. At that period, the inhuman treatment to which lunatics were subjected in public and private asylums had roused the active intervention of many benevolent persons. An extensive inquiry took place; and the state of Bethlem underwent a close investigation. The evidence taken upon that occasion revealed mismanagement so shameful, and scenes so revolting, that the public might reasonably have expected that the corporation, with so damning a record of past misgovernment before them, would have laboured incessantly to obviate all occasion for future animadversion. Where so solemn a warning has been disregarded, the natural conclusion is, that there is something radically wrong in the constitution of the governing body. In 1814 the following account was given of a visit to the women's galleries:—One of the side-rooms contained about ten patients, each chained by one arm or leg to the wall. The nakedness of each patient was covered by a blanket-gown only; this was the whole covering; the feet even were naked. Many unfortunate women were locked up in their cells naked, and chained on straw, with only one blanket for a covering. In the men's wing, six patients were chained close to the wall; all were naked, except as to the blanket-gown, and without shoes. The patients were not classed. In the men's wing were about 75 patients, with two keepers and an assistant. One man, Morris, attracted particular attention. He had been fastened by a long chain, which, passing through a partition, enabled the keeper by going into the next cell to draw him close to the wall at pleasure. He was afterwards confined in the following manner: a stout iron ring was rivetted round his neck, from which a short chain passed to a ring made to slide upwards or downwards on an upright massive iron bar, inserted into the wall; round his body a strong iron bar about two inches wide was rivetted; on each side the bar was a circular projection, which being fashioned to and enclosing each of his arms, pinioned them close to his sides. This waist-bar was secured by two similar bars, which, passing over his shoulders, were rivetted to the waist-bar both before and behind. The iron ring round his neck was connected to the bars on his shoulders by a double link. From each of these bars another short chain passed to the ring on the upright iron bar. He had remained thus encaged and chained more than twelve years. We have been thus particular in the description of the mode of confinement of this miserable being, because we conceive that so horrible a picture must always have been present to the eyes of the governors, and ought to have served them as a perpetual incitement to improvement; to create a new Bedlam, and to efface the foul memory of the old from the public mind. Nor can they have forgotten it. The portrait of the unhappy wretch, with his iron rings and sliding chains, in his nakedness and his squalor, abject woe upon his countenance, holds a place in the work of Esquirol, where it stands a degrading and enduring stigma upon the asylum where such things could be, and upon the country where they could so long be tolerated. Deriving his information from observation at Bethlem, which he not unnaturally assumed to be a fair representative of the other asylums of the country, Esquirol recorded his opinion that chains were nowhere so much in vogue as in England. With regard to the confinement of patients, we think it instructive to draw attention to the following circumstances. Dr. Monro being questioned upon this subject, answered: "I have seldom or never been consulted upon this matter; I should rather think myself it might be owing to the opinion of the matron in respect to the women, and very likely the steward in respect to the men, exercising their authority, under the direction of the apothecary, I presume."

The medical treatment was on a level with the notions of restraint. At that time Mr. Haslam was the apothecary, and Dr. Monro, an hereditary name at Bethlem, the sole physician. It was the practice to bleed, purge, and vomit the patients periodically. Like many other things at Bedlam, this plan seems to have been hereditary. The physician said that had been the practice invariably for years, long before his time; it was handed down to him by his father; he did not know any better practice.

We will not inflict upon our readers or ourselves the mental torture of tracing, beyond the above authentic facts, the sickening details of the evidence laying bare the practices in operation at Bethlem in 1814. We have to observe the mode in which the governors manifested their zeal in removing the abuses then prevailing, and the skill displayed in defending their conduct. The Hon. H. Grey Bennet visited the asylum in May, 1815. The change that had taken place in the appearance of the patients was most striking; no man was chained to the walls: on the women's side, two only were chained by the hand. The hospital was clean and sweet; but a want of classification appeared to be most sensibly felt. Mr. William Smith, M.P., who had observed the state of Bethlem prior to 1814, on examining it in May, 1815, reported to the same effect as Mr. Bennet; nor could he learn that any other cause existed for the alteration which appeared, but the change of the steward and the matron, and "*the attention which had been excited by the visits of last year.*"

The attention of parliament having been drawn to the management of Bethlem, a select committee of governors had met in June 1814 to consider the question generally, and to inquire into the case of Norris. Referring, possibly for the first time, to the history of this patient, and the reasons for keeping him in the horrible condition described, they found a resolution, bearing date June 16th, 1804—viz., "It appearing to the committee that James Norris is a very violent and dangerous lunatic, and had several times done mischief to the servants; ordered that he be put into the iron apparatus prepared for him, and approved by Dr. Monro and the committee, under the direction of the medical officers." The committee found that no complaint had been made by the patient, either of pressure or pain, during the whole period of his confinement. It appeared to them to have been rather a merciful and humane, than a rigorous and severe imposition. They concluded their report by representing their decided opinion, "that no foundation whatever existed for the imputation which had been made, and that, on the contrary, the general management of the hospital, as affecting the health, the cleanliness, and the comfort of the patients, was of a nature creditable to the governors and others concerned in its administration, *and such as would not suffer upon a comparison with any other institution of a similar description.*" This was at a time when the philanthropic and enlightened labours of Pinel and Esquirol were known throughout the world; and when the York Retreat had, under Mr. Tuke, given the most convincing demonstration of the blessings attendant upon a system more scientific and humane.

And now that we have placed before our readers a sketch of the Bethlem of 1814, as revealed before a Committee of the House of Commons, and the Bethlem of 1851, as revealed by the Commissioners in Lunacy, we are enabled to throw a light upon the causes and influences which have perpetuated the abuses that provoked the recent inquiry, and which recent transactions could not alone supply. It will be perceived that the government of this corporate institution has always been most exclusive in its character. They have always rejected every form of parliamentary and public inspection. In 1845 as in 1814, they insisted upon an exemption-clause. They have, throughout, upheld the inconsistent authority of the treasurer over the medical officers. In 1851 as in 1814, the matron had the power of confining and liberating patients. In 1851 as in 1814, the general care of the patients was left, to a most criminal extent, to the discretion of the attendants. In 1851 the treatment of the patients—medical and moral—was as much behind the science and improved practice of the day as in 1814. A blind routine has been obstinately followed throughout. Nor can we trace the origin of any improvement of importance to the spontaneous convictions of the governors or officers. In 1814 attentive observers could detect some symptoms of amelioration in the treatment of the patients, but they were obliged to attribute that "to the attention which had been excited by the visits of the previous year." In 1851, too, some reforms were introduced; but that, also, was, to use the graphic expression of one of the witnesses, not until after "things had a little bit of a stir." Experience had long proved that a more humane system of treating the insane, by diminishing the use of instruments of coercion, was more conducive to their safety and their cure; but the physicians could not be induced to adopt these innovations until threatened by Mr. Northall Laurie, that he would take means to make such a state of things public. The Dr. Monro of 1814 was never consulted upon the matter of restraint; he thought it might be owing to the matron in respect to the women, and very likely the steward in respect of the men. In 1851 we find the present physician of that name, while admitting that his authority as a medical man is not at all restricted, acquiescing in the printed and published rules of the hospital, expressly sanctioning the power of the matron to confine, and to liberate, and to distribute patients at her discretion! Experience had also proved that the protection and recovery of the patients could not be effectually provided for in a lunatic asylum unless by entrusting them to the supreme authority of a resident medical officer; and yet we see the governors of Bethlem maintaining, at a large salary, two physicians to make "formal and superficial" visits, for the purpose of destroying the responsibility of the resident medical officer. When we see them, therefore, appointing a resident physician with the necessary authority in the year 1852, *after* the inquiry of the commissioners, we cannot place much confidence in the sincerity of their conviction, that they had long before seen the necessity of adopting this measure; and we must accept with some hesitation their assurance that they had formed the intention of carrying out all the reforms that have since been forced upon them before the period of the inquiry. We think the whole history of the institution leads to the conclusion, that every reform of importance has been the result of external pressure. The close sequence of reforms upon occasions of public observation, is a fact that detracts considerably from the merit of introducing them.

The commissioners have pointedly called attention to the characteristic feature of Bethlem as an asylum, viz., that it is pre-eminently a hospital for the *cure* of insanity; and the remarks they make clearly reveal their opinion, that the construction and other arrangements of that institution are very ill-calculated to promote that object. Viewed in reference to this purpose, the defective arrangements and bad management prevailing throughout every department assume a far more serious import than would otherwise be the case. Need we remark that a *recent* case of mania requires a far more watchful and careful treatment on the part of the physician and the attendants than does a chronic case of dementia; that an error in classification, a harsh word, delay, or neglect in the treatment, the appearance even of a want of sympathy towards the recent maniac, may frustrate all hope of cure. If this be so—and what physician of judgment and experience will deny it—in what terms are we to express our condemnation of such a system as that which prevailed in Bethlem; where, on the male side, the patients were subjected to the most brutal violence; where, on the female side, the classification, distribution, and moral treatment were entrusted to a matron; and where, on both sides, reigned disorder and neglect? Who can calculate the amount of mischief that has been thus inflicted? Who can estimate the numbers of unhappy beings in whom the dawning ray of returning intellect has been rudely crushed and for ever extinguished? Who can estimate the number of those who have been driven headlong down the steep and fatal incline which leads from the curable to the incurable stage, and who have been prematurely thrust from the upper wards into that terrible basement, too surely and too soon reaching the next and last abode of misery—the incurable wards—whence all hope has fled, where all that remains of the long-tortured mind, is a shattered wreck!

There is one defect in the arrangements of Bethlem which has escaped the observation of the commissioners, but which we, remembering one of the leading objects of this Journal, cannot suffer to pass over without animadversion. A great deal has been said about the usefulness of Bethlem in promoting the knowledge of the nature and treatment of insanity. The governors have, of late years, professed a very praiseworthy desire to render Bethlem an efficient school for the study of mental diseases. We are compelled to say that in this object, as well as in their more immediate duty to the patients, the governors and the physicians have most lamentably failed. This institution, which from its metropolitan position, its ample resources, and the peculiar preponderance of recent cases, offered pre-eminent facilities for the most successful investigations in mental pathology, has been barren—literally and disgracefully barren—of any valuable results. No records, no detail of the character and progress of the cases under treatment, which are worthy of a moment's attention; no original contributions to psychological science, such as might have been expected from the long experience of those having the medical charge of the asylum; no published account of the morbid anatomy of insanity (excepting a few valuable papers contributed to this Journal by Mr. Holmes Coote), as the result of that rule which assigns to the surgeon the duty of inspecting the bodies of all the patients who died in the hospital. Bethlem has contributed nothing to the store of medical knowledge to compensate us for this oppressive load of odium and disgrace.

With regard to the provision made by the governors for the practical instruction of pupils, it strikes us as a remarkable circumstance, that although pupils have now for some years been admitted to the wards, the arrangements of the hospital, the gross neglect and ill-treatment of the patients, the absurd confusion of duties among the officers, the defective appliances which have so long constituted the normal condition of Bethlem, have not been before revealed to the public. Are students shown every part of the hospital? Are they conducted to the basement wards? Have they been shown how the refractory patients are treated in that dreary region? Have they day by day witnessed delicate women littered down like animals in straw, and have their lips been sealed?

We entertain a strong conviction that the proper association of pupils in the duties of a lunatic asylum, whilst it affords the most valuable aid to the physician in keeping the necessary records, and in the successful treatment of the patients, presents at the same time the most effectual security for the zealous discharge of the duty of the physician, both as regards his relations to the patients and the cultivation of science. The mere routine of "walking the hospital," as it has hitherto been carried out at Bethlem, and the subsequent monotonous humdrum of lectures, venerable from their antiquity, and tedious for the same reason, *read* to the students, form a system of instruction which falls as far short of the admirable practical teaching of a Baillarger, as does every other department of the hospital of the rapidly progressing improvements of the day. The only effectual method of instruction is, to introduce a limited number of internal pupils to assist the resident physician in his daily duties; these internal pupils, or clinical assistants as they might be called, should be selected from the external pupils after they have been sufficiently trained by clinical observation and *clinical* lectures. We believe no arrangement of this kind has been made; and we greatly fear the governors have not got the requisite *personnel* for carrying such a system into effect.

The past history of lunatic establishments has clearly proved the necessity of a well-ordered system of inspection, such as that now exercised by the Commissioners in Lunacy, as the only effectual means of repressing the growth of abuses, and securing humane and efficient treatment to the insane. We suppose we must ascribe it to a feeling of corporate jealousy, or perhaps to a consciousness of integrity, and reliance upon their own zeal, that the authorities of Bethlem have always, at least up to the time of the recent investigation, manifested a determined reluctance to submit to that official inspection which the legislature had at different times provided for the purpose of regulating the lunatic asylums of the country. In the report of the Committee on Madhouses, in 1815, it is stated that the governors of Bethlem had succeeded in the previous session in obtaining a clause, while the bill was in the house, for a partial exemption from the provisions of the act; and that circumstance was stated by the Committee, as a special reason for directing the attention of the house to the evidence relating to that establishment. When the new lunacy acts of 1845 were before the house, some independent members, on whose intelligence the past history of Bethlem had made a deep impression, moved that the exemption clause should be struck out. *It was struck out* accordingly. But the governors, obstinately faithful to their traditional policy, outwitted the Commons; somehow the clause was restored in the House of Lords; and they again succeeded in establishing an odious right, pregnant, however, with a terrible retribution. Now smarting under the sense of public reprobation, and perhaps seeing at last that many of the abuses which have disgraced their management have been fostered under the pernicious influence of the secrecy they had clung to, the opinion of the governors seems to be undergoing some modification. Mr. Northall Laurie plainly admits to the Commissioners that "the exemption of the hospital from the official visitation of the Commissioners has not been attended with advantage." Lord St. Leonards, in his recent exposition of his projected Chancery reforms, specially referred to the Bethlem exemption clause, as one of the abuses it was desirable to remove. We may conclude that at last it is doomed. It is not probable that the governors or the physicians will again be entrusted with the confidence of the Government—a confidence which they have signally abused.

But, having secured this exemption from external control, we may naturally inquire what internal arrangements the governors and the physicians had made to secure the ends which the official visitation of the Commissioners was designed to accomplish. There were two great objects which it was sought to attain:—1st, Due care of and attention to the comfort and cure of the patients. 2nd, A satisfactory guarantee that the public liberty should be respected. On perusing the evidence, on searching the printed regulations of the hospital, we unhesitatingly affirm, that we find no provision in which the slightest confidence can be placed. The Commissioners in their report specially observe, that the physicians never visited the hospital at night; nor did the treasurer, nor the committee. The visits of the physicians were of so "formal and superficial" a character, that it is next to impossible that the patients could have derived from them any valuable assistance towards their cure, or any security that they should not be improperly detained. The regulations of the hospital were such—regulations for which the governors are responsible—that the prospect of hearing the free complaints of patients of any ill-usage they might have received was hopeless. On the female side, no one—not even the apothecary—was permitted to visit the wards, unless in company with the matron or an attendant. There appears to have been no one specially charged with the duty of receiving complaints; and, for aught we learn, it was almost a matter of accident, whether persons who had recovered their reason obtained their discharge. If they did so, it was scarcely owing to the personal inquiry of the medical officers; a "formal and superficial" attendance could not be sufficient for that purpose. It was not owing to any inspection of the Committee; they may have walked round the wards once or twice a month, when everything was prepared for their reception; and sometimes the treasurer, in his own proper person, represented the whole committee. And this was the treasurer's notion of his duties in this respect. He never saw a patient without the attendant being present; he "did not expect he should hear more complaints if he were by himself; he made no inquiries specially of the patients; he heard everything the attendants were disposed to say."

The impression, the most detrimental to the well-being of a patient—the most fatal to any hope of obtaining his confidence, and of promoting his cure, is the feeling on his part, that there is no adequate protection for him against ill-treatment from the attendants, and no security against improper detention. Such a position is destructive of all cordial feeling between the physician and the patient. We know, indeed, that many inmates of Bethlem have felt, and felt keenly, this absence of any independent authority, to whom they could open their grievances; and this feeling, but too well grounded, has necessarily been a constant source of irritation and despondency.

We think there can be little doubt that a case is made out for placing Bethlem under some more stringent system of external supervision.

The recollection that the inquiry into the management of Bethlem cannot well stop with the report of the Commissioners in Lunacy, but that some further investigation is in all probability impending, which will terminate in the remodelling of this institution by a higher power, renders any detailed discussion of the efficiency of the late alterations made by the governors a much less important question than it might otherwise have been. But we cannot help observing, that, with all their experience of past errors, the governors have not yet made known to the public what measures they may have determined upon, to replace the hitherto repudiated official inspection of their asylum. We have been told by the president, with no small ostentation, that they have recently appointed a resident superintendent-physician of "great experience;" and we are assured that, under the care of that gentleman, Bethlem will be looked upon by foreigners as a "model asylum." Had the worthy alderman informed the court of the gentleman's age, he would have added immensely to their respect for his "great experience," by exciting their wonder at the incredible activity and grasp of mind which could accumulate experience without the usual sacrifice to inexorable Time. He did not, of course, think it necessary to remark, that, lacking the stamp of corporate affinity, the eminent qualifications and European reputations of other candidates failed to be appreciated by the governors.

Admitting, as we do, the weight of the experience the governors have acquired from their hereditary connexion with Bethlem; believing in the frequency of the hereditary transmission of insanity, and other forms of folly; recognising the fact of the hereditary transmission of corporate rights, and possibly of corporate virtues, we must be permitted to doubt whether the highest amount of eligibility for the office of physician to their asylum could be transmitted unbroken through three generations; and still more do we call in question whether civic magnates can transmit to their posterity, along with those virtues and talents of a specially civic character, any special aptitude for the acquisition of medical science, or the smallest tittle of ready-made experience. And, if not acquired by inheritance, where was the "great experience" of the new resident-superintendent obtained? Reckoning from the cradle, it is an experience of less than thirty years.

Yet, undoubtedly, the governors have given one very undeniable practical proof of the unlimited faith they repose in the "great experience" and extraordinary capacity of their protégé. In the ardour of their zeal for the reformation of abuses, they have heaped upon him work without stint and without measure. So large are his duties, that we cannot hope, within reasonable limits, to give them in outline; but we may convey some faint idea of their extent, by stating that his instructions occupy twelve pages of type. The committee who sanctioned these instructions must have been sadly oblivious of recent experience. Did they not recollect that Dr. Wood excused the charge of neglect alleged against himself, by pleading that he was taxed beyond his powers? and that the Commissioners admitted that there was some justice in the plea? Should not this example have warned them of the danger of a similar result?

But we are to have a triumphant reply.

The Court Circular informs us, that the president and treasurer have presented their reply to the Report of the Commissioners to the Home Secretary; and Mr. Walpole, in his seat in the House of Commons, has stated, in answer to a question from Sir Robert Inglis, that after having read that reply he should submit to the house the course he should recommend to be taken. It is not, therefore, probable that we shall be in possession of a copy of the governor's defence before going to press. But we are not altogether at a loss to conjecture what line of defence will be adopted. The president, in a speech before the Court of Aldermen, characterized by remarkable caution and reserve, complained that the investigation of the Commissioners was *ex-parte;* and that the governors had no opportunity of being present, or of cross-examining the witnesses. The governors will also probably expatiate upon their anxious solicitude to fulfil the great trusts committed to their charge; they will refer to the antiquity of their institution, and the amount of good it has been the means of effecting; they will probably admit that the constitution of the governing body has some defects, but they will take credit to themselves for having done their duty in adopting all necessary reforms; and they will endeavour to show, that while there has been much exaggeration and colouring as to the particular cases of neglect and cruelty brought before the Commissioners, the fault and the responsibility attaching to those cases rest with the medical officers and attendants.

Now, we are compelled to admit, that the evidence justifies the censure passed by the Commissioners upon the conduct of the medical officers; and we may make, as we do, a large allowance for exaggeration, but still we find it difficult to exonerate the governors. With them lay the chief power, and they must be content to bear at least a portion of the blame.

We trust that, for once, the governors will disregard traditional precedents, and that their present defence will not, like their former one in 1814, consist in a justification of the revolting practices brought then to light. We trust that they will not in 1852 exhibit the same audacity as in 1814, by appealing to the general opinion of the country, which they then said had for three centuries borne testimony to the superior claims of Bethlem to public estimation. We trust that they will break up the stereotyped formula employed by themselves in 1814, asserting that nothing in Bethlem "would suffer in *comparison with any other institution of a similar description.*" The physicians have adopted that in their letter of the 30th of April last: the governors must take up a different ground, and leave to these to maintain, as best they may, the burthen of proving that "the condition of Bethlem will challenge *comparison with that of any similar institution!*"

Casting an impartial glance over the past history of Bethlem, and perceiving what slender claims the institution has made out to the regard of the scientific community, or to the public gratitude, we entertain a less flattering opinion regarding it. We would wish to disabuse the minds of the governors of the fallacy under which they appear still to labour; whilst they have gone on, from generation to generation, congratulating themselves upon the success attending their pertinacious adhesion to traditional precedents, public opinion has been as uniformly transmitted in like manner in the opposite channel. We find it hard indeed to believe that the usually sound and discriminating judgment of the public should be recorded in favour of an institution remarkable for hugging obsolete forms and privileges, and which, the better to conserve them, has ever jealously excluded all public observation. The public confidence is seldom accorded to those who carry on their transactions in the dark.

And now we think the conclusion is inevitable, that the case of Bethlem stands out as a monstrous exception; that the shameful abuses, the culpable neglect of duties by the officers, the revolting cruelties of the attendants, unheeded by or unknown to the authorities, were the necessary and logical result of the hereditary self-perpetuating system of internal government, the errors of which, like rank weeds in noisome darkness, have flourished unrepressed under the protecting shadow of the exemption clause. We hope and believe that there is not now in England another asylum where such disorder, such abominations as have rendered the very name of Bethlem a synonym for all that is foul, corrupt, and humiliating, can be found.

A model asylum! Every physician, every Englishman, will scornfully protest against such a slander upon the national character. The form of government, the treatment of the patients, general, medical, and moral, have long since been repudiated and abandoned by every right-thinking man. If Bethlem be indeed a "model asylum," it is a model of the asylum of half a century ago; a warning model, set up, like the drunken Helot before the Spartan youth, to strengthen our admiration of what is good by the hideous exhibition of evil.

What steps the ministry will recommend to Parliament in the prosecution of this matter we are unable to determine; but we are at liberty to express the unhesitating conclusion which the past history of Bethlem and recent events have forced upon us. Our conviction is, that Bethlem, as an institution for the insane, is *three hundred and fifty years too old.* An extensive acquaintance with the actual condition of the lunatic asylums of this country authorizes us in asserting, that few institutions whose origin dates even from the beginning of this century have evinced a very cordial disposition to adopt the numerous improvements of modern times. Worn-out associations and prejudices cling with a morbid tenacity around the older buildings, and where they have been dispelled, it has mostly been owing to favourable circumstances, which have made way for the introduction of some physician whose education has been acquired, and whose sympathies have been nurtured, in a freer and healthier atmosphere. At the time even of erecting the new Bethlem in St. George's Fields, the governors were warned that the site was ill-adapted for the purpose; they could not prevail upon their own medical officer, Dr. Haslam, to attest its salubrity. At that period Bethlem scarcely supported 150 patients; now they surpass 400 in number. The whole area does not exceed eight acres, and the building itself takes up a large proportion of that limited space. Nearly 700 persons have within that space to seek the occupation, exercise, and recreation necessary to the maintenance of health! It would be an inestimable boon to the poor insane, it is a debt due to the humanity and enlightenment of the age, and it would be a lasting honour to the governors, to pull down the present building. The name of Bethlem, with its associations of loathing and terror, should be consigned to oblivion, and its ample funds should be bestowed in erecting a new state asylum in a healthier spot, under a new name and widely different arrangements. "Old Bedlam" might then give birth to an institution which we should not be ashamed to submit to the inquiring scrutiny of some future Esquirol, as an honourable example of the humanity and of the enlightened science presiding over the asylums for the reception and cure of the insane in the British Empire.

---

Since the above article was in type, we have been put in possession of "*The Observations of the Governors upon the Report of the Commissioners in Lunacy to the Secretary of State on Bethlem Hospital.*" We are anxious to give the governors and the medical officers the full benefit of any reply they may consider it necessary to make to the serious charges alleged against them. The "Observations," however, extend over one hundred and seventy pages, and even abstracting a large amount of correspondence, and totally irrelevant matter, we cannot find space for the insertion of the replies of the governor and of the physicians in detail. Indeed, giving to these documents the most careful consideration, we do not think it would much promote the object proposed if we were to print them entire. Still we feel it a duty to make such references to the facts and arguments urged in their replies as may be useful in elucidating the subject. The governors, in terms betraying considerable irritation, dispute the authority of the commissioners, and accuse them of partiality in their conduct of the investigations.* The governors again protest that the most important charges rest upon evidence which would be excluded from ordinary tribunals; they assert that an unsparing use was made of leading and suggestive questions; they complain that the governors and other parties implicated were not allowed to be present, and that the witnesses could not be cross-examined; they submit that it was the obvious duty of the commissioners to communicate the specific charges in the presence of those who made them; and they find it difficult to imagine how the commissioners could feel themselves justified in arriving at any conclusion whereon to base a report without those explanations and answers a different course would have afforded them. They admit, indeed, that the president and governors received an invitation from the commissioners to make any statements they might wish to submit, but they think they could not do otherwise than decline to make "any statement" so long as the governors were "unacquainted with the wishes of the commissioners and *of what occurred before them.*"

It is impossible to deny that there is some justice in the complaint of the governors. They ought to have had the opportunity of hearing the depositions of the witnesses; and we think they could take no other course than refuse to make any statements to the commissioners *in ignorance of the charges brought against them, or of what had transpired at the official inquiries.* But we fear that it is in those technical objections, however important in principle, that the main strength of the governors' reply consists. When they seek to justify the past regulations and mismanagement of the hospital, or to rebut the specific charges adduced, they meet with little success; and they moreover involve themselves in the most palpable contradictions. And although they may succeed in creating a belief that the commissioners did not adhere rigidly to the usual forms of judicial inquiries, the governors will not, on *that account,* be enabled to satisfy the public that the evidence taken does not bear out the report based upon it. In reference to one of their objections,—an objection which they frequently recur to for the purpose of invalidating the conclusions against them,—the governors should be reminded that the testimony of persons *who have been of unsound mind* is not inadmissible in courts of justice; and that if the

* Mr. Johnson, the treasurer, remonstrates "against the mode in which the evidence was taken, as un-English in its character, and at variance with those constitutional principles which are respected and recognised in this country."

commissioners had seen fit, they might, warranted by recent precedents, have gone further, and have *examined persons actually insane.* Of course evidence of this kind must be taken with much circumspection; but we think all humane and judicious persons are agreed that if it were on all occasions excluded, we should be deprived not only of a valuable aid in the discovery of truth, but of a most effectual check against oppression. And after all, that which really concerns the public is, whether the charges made against the government of Bethlem are so far supported by trustworthy evidence, as to show that it could not, safely or consistently, be left uncontrolled in the hands which have hitherto wielded it?

Entering upon the merits of the case, the governors submit that the evidence does not bear out *many of the most important* of the *conclusions* arrived at by the commissioners. They confine their attention to the *five cases* reported upon by the commissioners; and appear plainly to intimate that five cases can afford no satisfactory grounds for condemning the management of an institution admitting about three hundred and forty patients annually. To this it may be answered, that if the treatment adopted at Bethlem is in accordance with certain rules, and is part of a system, the treatment experienced by five patients, or even by one, is amply sufficient to illustrate the system pursued. But the governors proceed to contend that the charges arising out of these five cases are not fairly established. We will follow them consecutively, but briefly. In Miss A. M.'s case the main facts as to the deplorable condition in which she left the hospital are quite untouched by the governors' observations—they rest indeed upon unimpeachable testimony—but they attempt to throw discredit upon her statement that she had no night-clothing, and had insufficient bedding. They remark: "*It is not to be credited that this patient had not at night the usual supply of bedding seen in these bed-rooms every day.*" Why is it not to be credited? Did they ever visit the rooms *at night* to see? We submit the following passage from the Annual Report of the Commissioners to the Lord Chancellor for 1850:—"Having felt it our duty to comment on the objectionable practice in two other establishments *of placing sheets on certain straw beds during the day, which were removed at night*, we regret to say that we discovered that such a practice existed also at St. Luke's *without the knowledge either of the resident medical officers or the visiting physicians.*" The argument of the governors of Bethlem is not, then, supported by experience, and it certainly cannot be commended for logical consistency.

The case of Miss H. H. is passed cursorily over. The governors dwell chiefly upon the want of evidence to show that her death was the result of ill-treatment received in the hospital; they refer to the evidence of Sir A. Morison and Dr. Wood, and to the certificates respecting the *post-mortem* examination by Drs. Taylor and Crisp and Mr. Lawrence. The evidence of one of the physicians upon this subject is as follows:—"I found several superficial bruises on different parts of her body, and redness and tendency to excoriation about the nates, none of them at all connected with the cause of her death, which was occasioned by general paralysis. *The bruises I consider to be accidental, and in no way to be attributed to want of care and kindness.*" The pathological acumen displayed in these conclusions is somewhat remarkable, and our professional readers would probably desire to learn upon what grounds he was enabled, from the inspection of the sores and bruises, to decide that they could in no way be attributed to want of care? The certificate of Mr. Lawrence, although strangely interpreted in a different sense by the governors, can leave no doubt whatever that poor Miss H. had suffered deplorably from want of care and cleanliness whilst in the hospital.

The third and fourth cases, those of Mrs. Elinor W. and Mrs. Mary Elizabeth W., were referred to by the Commissioners as having been subjected to harsh and improper usage from the attendants, and that they were neglected in the hospital. The governors are at some pains to show, that the charge of "mopping" the dirty patients rests upon the sole evidence of Mrs. Elinor W.; we may be excused from following them in this argument, because the Commissioners *do not bring forward this charge in their conclusions.* The case of H., in addition to the general charge of ill-usage made by the Commissioners, involves the imputation, also *not referred to* in the conclusions of the Commissioners, of using the stocking after the manner of the "garrotte." The governors submit, that the evidence upon this point resting mainly upon one witness, John Welsh, a rejected attendant, is untrustworthy. We hope it may be so; but the governors have failed to submit anything to convince the public that, if such a practice did not exist in Bethlem, there existed any security to obviate that or any like atrocity on the part of the attendants. We altogether dissent from their proposition, "that governed and constituted as Bethlem is, it is impossible for any system of unkindness to be practised there without early detection."

The governors, who throughout their observations seldom neglect an opportunity for an interjectional sarcasm against the Commissioners, excuse themselves for not "visiting at night" on the ground of the *very sparing use of this delicate part of their authority* by the Commissioners themselves, who, the governors take care to remark in another place, are *paid* for the performance of their duties; but in justice to the Commissioners it should have been stated, that they are not empowered to make night visitations to any asylum, unless they have before them evidence upon oath of the existence of practices requiring their immediate attention.

With regard to the want of infirmaries, the governors appear in a rather singular position. They first carefully point out how unnecessary infirmaries are in Bethlem, where most of the patients have separate rooms, and then inform the Home Secretary that buildings for new infirmaries are nearly completed! Notwithstanding this and many other contradictions equally striking, of their own, they devote a page to a severe lecture to the Commissioners for their dulness in ignoring the "broad and clear distinction between a '*physician*' and a '*visiting physician.*'" We have laboured with some earnestness to find out in what manner the conclusions of the Commissioners are vitiated by this unpardonable error; but as the governors have neglected to make any application of their discovery, we may be excused for not perceiving its importance. We leave it to the "physicians" or "visiting physicians" to appreciate this chivalrous vindication of their proper professional titles.

We may observe, that the general committee have not a word to say in defence of the imperfect state of the records.

We may most conveniently consider the remainder of the "Observations" as the individual replies of the treasurer, of the medical officers, and of the matron. The treasurer, our readers will remember, was charged with having injudiciously invested the matron with the power of classifying the female patients, and of regulating their employment. The committee, *first, deny* that he did so. They refer to the rules to show that these are amongst the most important duties of the *physicians.* The apothecary could only act in the absence of the physicians; therefore, we presume, the treasurer, in interdicting the interference of the apothecary, did not touch on the province of the physicians! But, *secondly,* the treasurer, "by confirming the matron in her peculiar duty (which of course embraces the duties referred to), secured an increase of employment, and increased advantages to the patients. The governors entertain no doubt whatever of the propriety of the course the treasurer pursued." After this unequivocal defence of the propriety of subjecting the moral treatment of the female patients to the care of the matron, we find it somewhat difficult to account for that instruction to the matron in the *revised* rules, which says, "she shall not remove any patient or attendant from one gallery to another, without the authority of the resident physician." As in the case of the infirmary, they pertinaciously justify the old course, and straightway adopt the opposite!

The reply of Dr. Monro it is difficult to characterize. Instead of an earnest vindication of his conduct, such as might have been expected from one who had been charged with the most serious neglect of duty, and indifference to the claims of his profession, and who at the same time felt that he was wrongfully accused, we are favoured with a description of the writer's *beau ideal* of the "mental physician," and some remarks upon the evidence in a tone not to be commended, and having little or no bearing upon the points at issue. Dr. Monro's estimate of the duty of the physician is thus described: "to pay certain periodical visits, and to exercise a large and superintending control over the medical treatment and general management of the hospital, and by no means to include the regulations of details which must attach to subaltern officers. Indeed, such points as these never have constituted any part of a physician's duty. The modern idea appears to incline to that hard-working attention to *minute particulars* which has *never hitherto characterized the mental physician* exercising a high profession in a liberal manner; and if the duties of the future medical officer are to be so minute, and so extensive, and so laborious, he must indeed be of a very different grade and calibre from all physicians who have hitherto exercised this high calling." We cannot, of course, presume to attach any censure to a physician who stands upon such a lofty professional pedestal. We can only regret that this picture of the "mental physician" has but few admirers in this utilitarian age. This attention to "minute particulars" constitutes an important feature in the psychological physician of *modern* times, as compared with those born and bred amidst all the prejudices and obsolete notions of the last century. What can be done in the successful treatment of insanity if the attention to "minute particulars" is not, in all cases, enforced? But as Dr. Monro does not disdain to throw himself upon the governors, we will let the governors answer him:—"They cannot conceive how any physician could exercise a large control, or could superintend and direct subordinate officers, without giving that amount of attention to minutiæ and details absolutely necessary to make himself acquainted with at least all the ordinary proceedings in the institution under his care."

Dr. Monro thinks he can escape from the dilemma of having acquiesced in the proved systematic ill-treatment of the patients, or of having neglected his duty, by such affectation as the following:—"For instance, it might be suggested—Did you not know that a certain patient was fed by the keepers with brimstone and treacle? I never saw anything of the kind.—But do you not, in your capacity as physician, know that such was the case? Indeed I do not. Why here, gentlemen, is a medical officer of thirty years' standing who does not know whether it be true that a certain patient lived on brimstone and treacle?" It is not thus that so serious a charge should be paltered with.

With reference to the defective reports, he thinks it sufficient to say, that "they are, *of late years*, prepared with much care, and contain all necessary information;" and he has, *therefore*, just reason to complain of Mr. Laurie's evidence.

Dr. Monro does not deem it beneath him to remind the governors of his long services and hereditary connexion with Bethlem. "For myself, after thirty-six years of unremitting attendance, I may be permitted to speak of the institution and its inmates with the deepest regard. Nobody can pretend to the same amount of interest—preceded, as I have been, by several of my immediate ancestors through a period of, altogether, one hundred and twenty-five years."

——— Hæres

Hæredem alterius, velut unda supervenit undam.

But if Dr. Monro fails himself to grapple with the direct charges brought against him, he displays considerable astuteness in throwing his burthen upon others. It is impossible not to sympathize in his appeal to the governors to defend him, and to admire the skill with which he makes them parties to the charge. Dr. Monro observes—"The condition of Bethlem Hospital has been, in fact, a subject of encomium for years, as is incontrovertibly verified by the uninterrupted succession of approbation contained in the written observations of the *governors*. These books alone proclaim the impression of half the world, and of many of the governors themselves, and are a clear justification of the past. Let me say, in conclusion, that whatever course the governors may think it right to adopt in other matters, it is quite clear that officers who have so long enjoyed the countenance and approbation of the governors, must look to them for the defence and vindication of their professional integrity, which can alone be satisfactorily shown by some express minute to this effect."

After thus throwing himself upon the generosity of the governors, the following response seems a little ungracious:—"*The governors are sorry that Dr. Monro's concluding remarks prove the propriety of those changes in the medical staff that have recently been made!*"

It is true the committee have not carried their changes so far as to altogether discard the physicians, although they have thought it necessary to supersede them in the exercise of all that a physician usually deems useful or honourable. It is thus that Dr. Monro announces his resignation (not of his office, but) to his altered position:—"After so very long a service, the sudden transition to new modes, involving (to say the least) many modifications of my own duties, is, of course, very painful, and wholly unexpected." If Dr. Monro conceives himself to be sacrificed by the governors, it is his duty to himself, as well as to the profession, to resign at once all connexion with the hospital?

The reply of Sir A. Morison is a more painstaking and satisfactory production. He enters minutely into an examination of the charges against him, and shows, and not without some success, that the strictures of the Commissioners upon the physicians for their imperfect performance of their duty, do not apply so much as was alleged to himself. *He denies Dr. Wood's statement that he ever did duty for him.* He says, that he writes in his private case-book the history of every patient. He shows, that although the patient Miss H. H. was in his department, yet being called out of town, she was left under the care of Dr. Monro and Dr. Wood, and that he only returned to his duty two days before she was removed from the hospital. We refer to what we have previously said concerning Sir A. Morison's certificate in this case. There is not much in Sir A. Morison's reply which appears to call for special comment. He discusses the matter in a serious and candid spirit, and succeeds to a certain extent, as we have remarked, in freeing himself from the imputation of inattention to his professional duties. He does not, however, in the remotest degree, shake our conclusion that the constitution of the medical staff loudly called for revision.

Dr. Wood, the late apothecary of Bethlem, is not yet prepared with his reply. In a letter, dated the 6th of October, addressed to the committee, "he thinks it right to say that he cannot complete his observations within the time, and therefore begs the committee will leave him at liberty to make his answer independently of their report." As the case of the governors and the physicians is not much mended by their respective "observations," it is probable that Dr. Wood has exercised a wise discretion in his reserve.

The remarks of Mrs. Hunter occupy fifteen pages. They certainly evince considerable care; but we think the real defence of the matron (if a defence can be made) lies in this—that she was acting in obedience to the authorities of the hospital.

We find in the "Observations" some elucidation of the motives which led the governors to remodel the institution. In a letter, replying to an interpellation from the physicians, dated 7th May, 1852, they observe—"The appointment of a resident physician and medical superintendent, which was unanimously agreed to by the committee, and unanimously confirmed by the last court, had long been felt by the president and treasurer, and many governors . . . . . . to be a necessary and desirable alteration in the system pursued at Bethlem Hospital."

We, however, frequently obtain a more correct insight into actuating motives from the less guarded expressions of individuals than from more studied official communications. The treasurer, in answer to a letter from Dr. Monro, had, ten days previously to the foregoing statement of the committee, made the following admission :—" Recent events have rendered it imprudent to delay any longer the re-arrangement of the medical staff!"

In conclusion, we may—without, after our lengthened survey, dwelling upon other omissions—remark upon one most important defect in the "observations of the governors." They have altogether neglected to state what provisions they had made in substitution of the official inspection of the Lunacy Commissioners which they had rejected. They have boasted that their exemption from this control was a mark of the *confidence of Government* (!) : it certainly threw upon them a most important and undivided responsibility. Their failure in this imperative duty to the public is alone a sufficient ground for the abolition of an odious privilege.

The "observations" of the governors will certainly fail to convince the world that the report of the Commissioners in Lunacy was based upon insufficient evidence. They are not conceived or put forward in a spirit that will gain them sympathy; and they will certainly not meet with approbation on account of their clearness or consistency. Like many productions made up from the contributions of different individuals, these "observations" betray continual clashings of opinion, and an unsteady adherence to any particular line of defence. At one moment we find the governors earnestly defending their past policy, and at the next taking credit for having reversed it. They seem ever at a loss to decide upon what ground to take their stand. The state of mind under which the "observations" were composed might be not inaptly illustrated by the following amended version of an old confession of mental indecision:—

"Video *deteriora* proboque, *meliora* sequor."

We hope they will continue faithful at least to the latter half of the motto.

---

